# Target trial emulation of carfilzomib safety among patients with relapsed/refractory multiple myeloma using a nationwide observational data in Korea

**DOI:** 10.1007/s00432-024-05800-8

**Published:** 2024-05-20

**Authors:** Hyun Kyung Lee, Ha Young Jang, In-Wha Kim, Jung Mi Oh

**Affiliations:** 1 College of Pharmacy, Seoul National University, Seoul, Republic of Korea; 2 College of Pharmacy, Gachon University, Incheon, Republic of Korea; 3 College of Pharmacy and Research Institute of Pharmaceutical Sciences, Seoul National University, Seoul, Republic of Korea

**Keywords:** Drug-Related Side Effects and Adverse Reactions, Multiple Myeloma, Proteasome Inhibitors, Administrative Claims, Cohort Studies, Asian People

## Abstract

**Purpose:**

Carfilzomib, commonly used for relapsed/refractory multiple myeloma (RRMM), has been associated with various adverse events in randomized controlled trials (RCTs). However, real-world safety data for a more diverse population are needed, as carfilzomib received expedited approval. This study aimed to evaluate carfilzomib’s safety in Korea by comparing new users of KRd (carfilzomib, lenalidomide, and dexamethasone) to Rd (lenalidomide and dexamethasone) using a nationwide administrative claims database.

**Methods:**

The retrospective cohort study utilized target trial emulation, focusing on adverse events in various organ systems similar to the ASPIRE trial.

**Results:**

This study included 4,580 RRMM patients between 2007 and 2020, and the KRd group showed significantly higher risks of hematologic adverse events (anemia, neutropenia, thrombocytopenia) and some non-hematologic adverse events (cough, hypokalemia, constipation, hypertension, heart failure) compared to the Rd group. Among non-hematologic adverse events, cardiovascular events (heart failure [HR 2.04; 95% CI 1.24–3.35], hypertension [HR 1.58; 95% CI 1.15–2.17]) had the highest risk in the KRd group.

**Conclusion:**

The safety profile of carfilzomib in Korean patients was similar to previous RCTs. Therefore, caution should be exercised when using carfilzomib in Asian individuals with RRMM due to the increased risk of cardiovascular adverse events.

**Supplementary Information:**

The online version contains supplementary material available at 10.1007/s00432-024-05800-8.

## Introduction

Multiple myeloma (MM) is one of the most common hematologic malignancies (National Cancer Center 2023), and its incidence is increasing with the aging population worldwide (Turesson et al. 2018). While it has traditionally been observed that the incidence of MM is lower in Asians compared to Western countries (Ludwig et al. 2020), recent trends show an increase in diagnoses among Asian populations. This growing number of MM cases in Asians, including in Korea where the incidence has doubled within a decade (Hong and Lee 2016; National Cancer Center 2023), emphasizes the need for further research and attention to this patient group.

In recent years, significant advancements have been made in the development of treatments for MM, including the introduction of proteasome inhibitors (Scheid et al. 2021). One such drug is carfilzomib, which is a second-generation proteasome inhibitor that received accelerated approval from the U.S. Food and Drug Administration (FDA) for the treatment of relapsed or refractory multiple myeloma (RRMM) (Herndon et al. 2013). Additionally, carfilzomib was approved in South Korea in March 2017 and has become the preferred treatment for RRMM.

While carfilzomib has demonstrated promise in enhancing patient outcomes, including progression-free survival, overall survival, and quality of life, concerns about its safety profile persist due to reports of significant adverse events such as heart failure in various clinical trials (Dimopoulos et al. 2016; Hájek et al. 2017; Stewart et al. 2015). Additionally, although no differences in the pharmacokinetics and metabolism of carfilzomib were identified between Asians and non-Asians (Ou et al. 2017), subgroup analyses of clinical trials reported a higher incidence of adverse events in Asians compared to the overall study populations (Dimopoulos et al. 2019). Nevertheless, the clinical trials of carfilzomib included only a limited number of Asian participants (Dimopoulos et al. 2016; Hájek et al. 2017; Stewart et al. 2015). These highlight the need for real-world safety studies of carfilzomib, especially among Asian populations that were underrepresented in randomized controlled trials (RCTs). To address these concerns and generate more substantial evidence, safety studies of carfilzomib were conducted in Japan (Kawaji‐Kanayama et al. 2022; Kawasaki et al. 2022) and Korea (Jang et al. 2022; Lee et al. 2021). Particularly, a previous study with a small cohort of 138 patients, which utilized the target trial emulation (TTE) method using electronic medical records of a tertiary hospital in Korea, identified significant increased risk of dyspnea, muscle spasm, and thrombocytopenia in carfilzomib users. However, these previous studies had limitations such as small sample sizes, lack of comprehensive data, or absence of an active comparator group, which limited their ability to provide conclusive evidence on the risk of carfilzomib. Therefore, this current study aims to address these limitations by analyzing the safety of carfilzomib using a larger sample size and Korean nationwide observational data. Through this study, the researchers intend to contribute valuable evidence regarding the safety of carfilzomib in Asian populations, specifically in Koreans, and enhance understanding of its risk profile.

In this study, the researchers utilized the TTE method to overcome the challenges associated with conducting an RCT to evaluate the safety of carfilzomib in Asian populations. The TTE method involves designing hypothetical RCTs that can be simulated using real-world data, providing a guide for conducting reliable observational studies (Hernán and Robins 2016). This approach has gained attraction in drug safety studies, with an increasing number of studies adopting this methodology (Ahn et al. 2022; Rossides et al. 2021; Takeuchi et al. 2021; Xie et al. 2020). By constructing a hypothetical RCT and emulating it using real-world data, this method enables more systematic analysis and allows for the interpretation of results similar to those obtained from RCTs (Zhao et al. 2020). Therefore, in this study, a retrospective cohort study was conducted within an RCT-like setting, utilizing a sufficiently large number of subjects. The purpose of this approach is to provide more clinically relevant information regarding the safety of carfilzomib and its use in Asian populations.

The objective of this study was to assess the safety of carfilzomib in the treatment of RRMM in the Korean patients and to evaluate if the results from the RCTs could be applied to the broader Asian population.

## Materials and Methods

### Data source

This study used the customized Health Insurance Review and Assessment Service (HIRA) data in South Korea from January 1, 2007 to May 31, 2020. HIRA data is a nationwide data including over 50 million people because almost all of the Korean population is covered by a single national insurance. This data includes information about patients’ demographic characteristics, diagnosis codes, and treatment (Kim et al. 2020). All patient information was anonymized. This study was approved by the Institutional Review Board of Seoul National University (IRB No. E2101/001–003).

### Study design

**Fig. 1:**
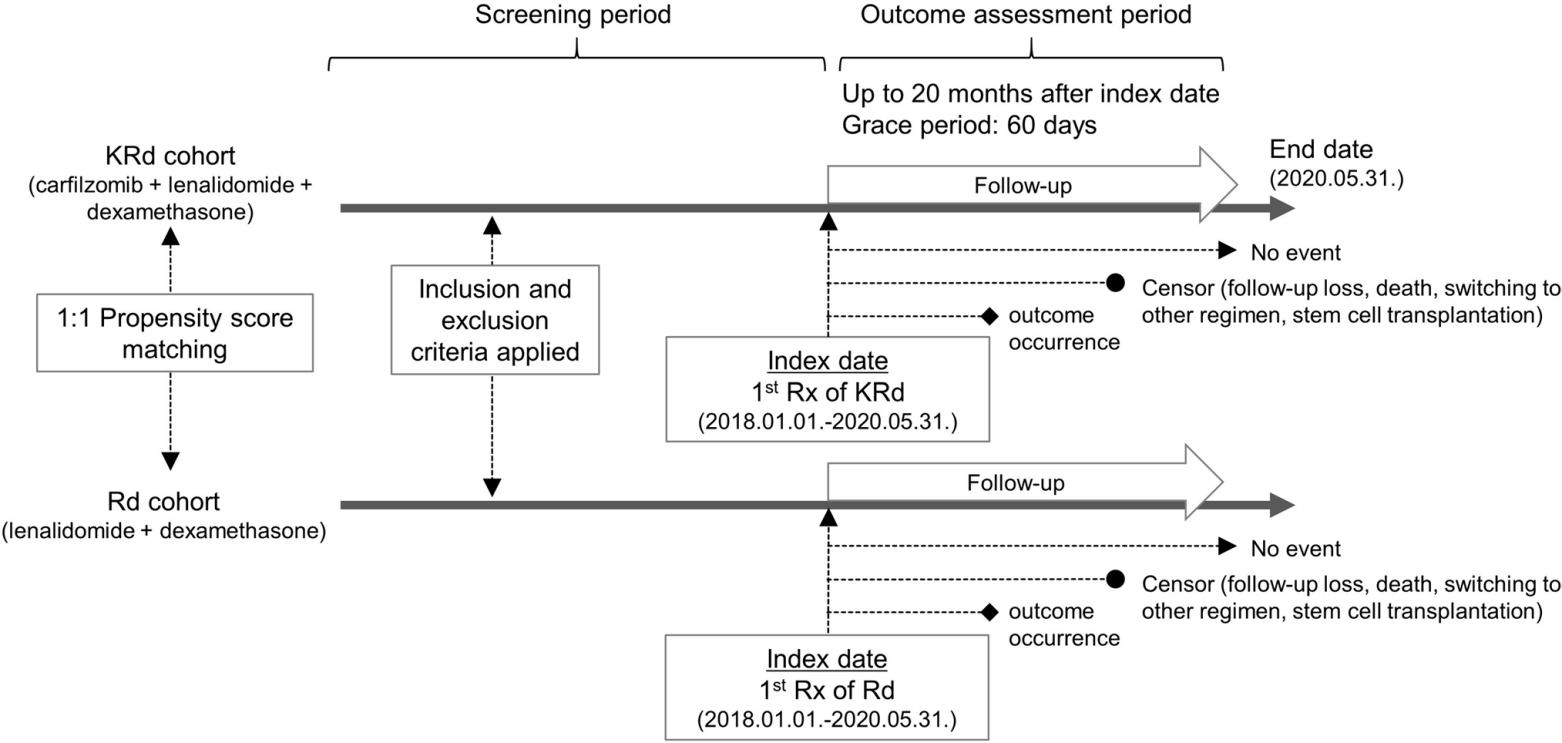
Study design to evaluate adverse outcomes of KRd or Rd regimens. Rx, prescription

This was a retrospective cohort study using a TTE method (Fig. 1, Table 1) modified with reference to the ASPIRE trial (Stewart et al. 2015). Individual cohorts were constructed for each outcome variable. The study followed the per-protocol method. The index date was set as the first administration date of the KRd (combination therapy comprised of carfilzomib, lenalidomide, and dexamethasone) or Rd (combination therapy comprised of lenalidomide and dexamethasone) regimens. Censoring dates were determined based on several factors, including the occurrence of the endpoint, the last date of data collection, 600 days from the index date, the study end date, 30 days from the last administration date, and the date of treatment change or stem cell transplantation. A grace period of 60 days was implemented, since the claims data provided for inpatient medical insurance may have a lag time due to monthly claim submissions.

**Table 1:** Study protocol of the hypothetical target trial and emulated trial using claims data

	Hypothetical target trial	Emulated trial using claims data
Eligibility criteria	Patients with relapsed or refractory multiple myeloma after receiving at least one chemotherapy No previous prescription of carfilzomib No prior history of the safety outcome variables	Same as the target trial
Treatment strategies	Patient group: KRd (carfilzomib 27 mg/m^2^ at Day 1, 2, 8, 9, 15, 16 + lenalidomide 25 mg at day 1–21 + dexamethasone 20 mg at day 1, 8, 15, 22) Comparator group: Rd (lenalidomide 25 mg at day 1–21 + dexamethasone 20 mg at day 1, 8, 15, 22)	Patient group: KRd (claims for carfilzomib, lenalidomide and tablets or injectable dexamethasone on the same date) Comparator group: Rd (claims for lenalidomide and tablets or injectable dexamethasone on the same date and no other anticancer drugs claims on the same date)
Assignment procedures	Randomized, not blinded	1:1 propensity score matching
Follow-up period	From the time of treatment assignment, up to 20 months The censoring criteria are the first outcome occurrence, death, switching to other regimens, stem cell transplantation, or 30 days after the last drug administration	Same as the target trial
Outcomes	Hematological adverse events (anemia, neutropenia, thrombocytopenia) Non-hematologic adverse events (diarrhea, fatigue, cough, pyrexia, upper respiratory tract infection, hypokalemia, muscle spasms, peripheral edema, constipation, back pain, dyspnea, peripheral neuropathy, hypertension, acute renal injury, heart failure, deep vein thrombosis, ischemic heart disease, pulmonary embolism)	Same as the target trial
Statistical analysis plan	Per protocol Continuous variables: mean ± standard deviation, median (range), Student t-test or Mann–Whitney U-test Categorical variables: frequency, incidence (events/10 person-year), hazard ratio (95% confidence interval), Cox regression analysis	Same as the target trial

### Study population

The study included patients diagnosed with MM between January 1, 2007 and May 31, 2020 and had been previously received at least one treatment regimen other than KRd or Rd. To select new users of KRd or Rd for RRMM, the exclusion criteria were as follows: patients previously prescribed carfilzomib, or those who received KRd or Rd as first-line therapy. Furthermore, different exclusion criteria and screening periods were used according for each outcome variable in various organ systems as outlined in Supplementary Table 1. By excluding patients with pre-existing outcome variables before the index date, the study aimed to establish cohorts that specifically focused on the association between the drug regimen (KRd or Rd) and the occurrence of the outcome variables of interest.

### Exposures

The KRd regimen was defined as the case treatment while the Rd regimen was defined as the comparator treatment. The exposure to the KRd regimen was defined as the presence of claims for carfilzomib, lenalidomide, and tablets or injectable dexamethasone on the same date. On the other hand, exposure to the Rd regimen was defined as the presence of claims for lenalidomide and tablets or injectable dexamethasone on the same date with no claims for any other anticancer drugs claims on that specific date.

In consideration of the reimbursement timeline for carfilzomib in South Korea, the exposure window in this study was set from January 1, 2018 to May 31, 2020. This timeframe was chosen to include the period when carfilzomib became eligible for reimbursement, ensuring that patients who initiated treatment with carfilzomib during this timeframe were included in the analysis. A maximum of 600 days from the date of first prescription was defined as the duration of exposure.

### Outcomes

The primary focus of this study was to examine adverse events in various organ systems that occurred with a higher frequency in the carfilzomib group compared to the comparator group, specifically with a difference of at least 5 percentage points and other adverse events of interest based on the findings from the ASPIRE trial (Stewart et al. 2015). The study included comprehensive set of outcome variables consisting of 21 different adverse events on different organ systems. These adverse events encompassed a wide range of organ systems including blood and lymphatic, cardiac, gastrointestinal, general, infections, metabolism and nutrition, musculoskeletal and connective tissue, nervous, renal and urinary, respiratory, and vascular disorders.

The diagnosis of MM and other relevant medical conditions was defined according to the Korean Standard Classification of Diseases (KCD) version 8. For drug utilization, the Anatomical Therapeutic Chemical (ATC) Classification System code was used. The procedures were defined using procedure codes, which provided a standardized method for identifying and classifying specific medical procedures or interventions. The operational definitions for each adverse event outcome variable are shown in Supplementary Table 2 with references. The operational definitions were referenced by integrating the definitions used in previous research methods. The outcome occurrence date was defined as the first occurrence date.

### Covariates

Basic patient information, comorbidity, MM treatments, and other medications were collected and included as covariates. The operational definitions and screening periods for the covariates apart from the outcome variables are summarized in Supplementary Table 3.

### Statistical analysis

In the individually constructed cohorts, the propensity scores were calculated using logistic regression. The outcome variable was excluded from prescreening process and the remaining outcome variables were matched as covariates. Two groups were matched 1:1 using the 5-to-1-digit greedy matching algorithm (Parsons 2001) in order to balance the covariates between the carfilzomib and comparator groups. A standardized difference of less than 0.1 between the groups was considered an indication that the confounding factors had been adequately matched.

Descriptive analysis was performed for both covariates and outcome variables. Continuous variables were summarized using either median (range) or mean (± standard deviation). Categorical variables were presented as the frequency and percentages (%). To assess the relationship between treatment and the outcomes, the hazard ratio (HR) with 95% confidence interval (CI) was calculated using the Cox proportional hazard model. Additionally, Kaplan–Meier survival analysis was performed to estimate and visualize the survival probabilities over time for the carfilzomib and comparator groups.

Two sensitivity analyses were conducted to evaluate potential biases and further investigate the robustness of our findings. The first sensitivity analysis involved changing the comparator group to historical comparators who had received the Rd regimen before January 1, 2018 to evaluate the potential bias resulting from changes in treatment guidelines over time. The second sensitivity analysis involved performing the analysis without limiting the grace period to assess the potential bias resulting from the grace period setting and explored its influence on the observed outcomes. For the statistical analysis SAS Enterprise Guide 6.1 and SAS 9.4 (SAS Institute Inc., Cary, NC, USA.) were used.

## Results

### Baseline characteristics

**Fig. 2:**
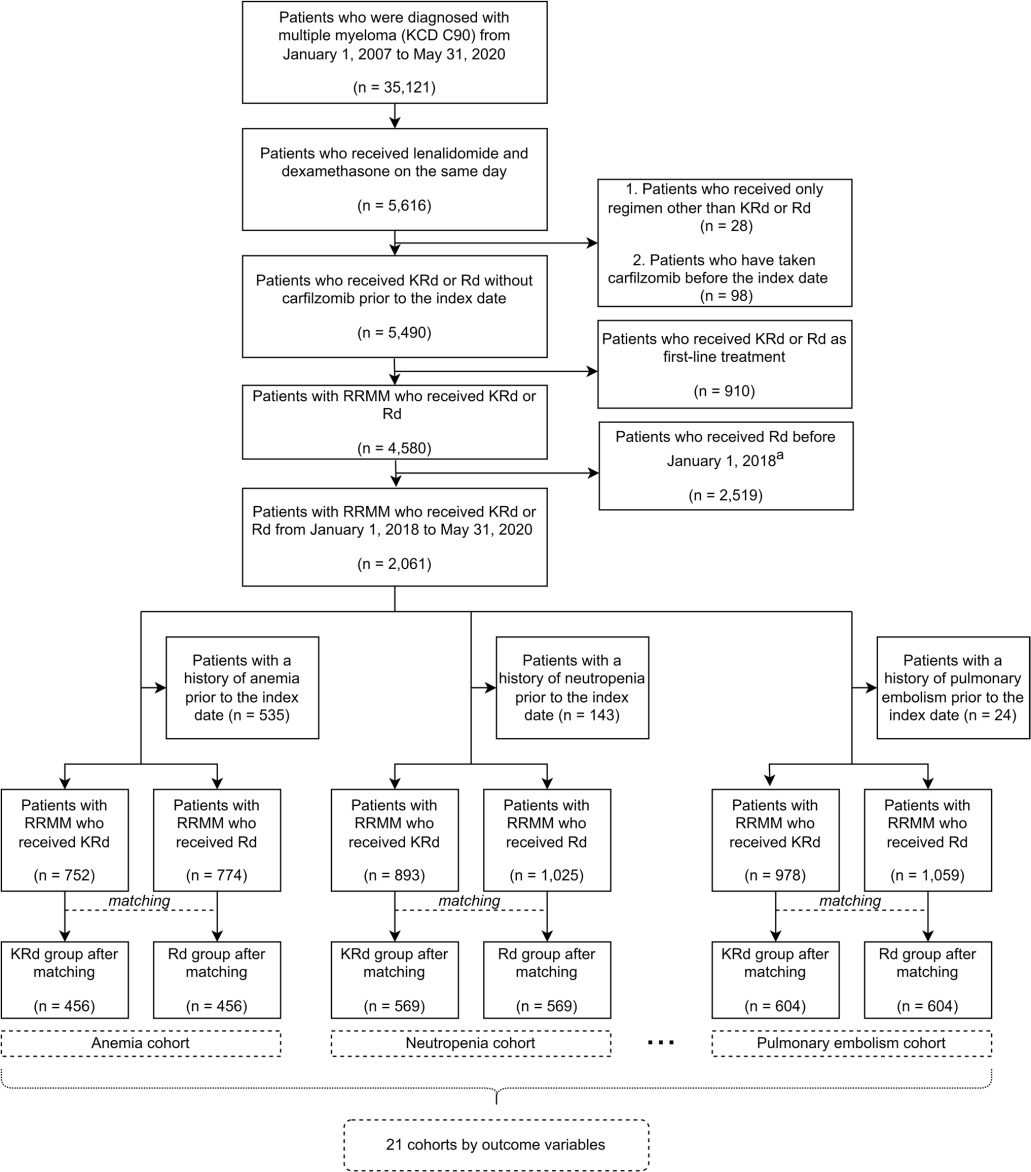
Flow chart of subject selection to evaluate 21 adverse outcomes of KRd and Rd regimens. The index date was the first administration date of KRd or Rd regimen. The outcome variables were anemia, neutropenia, thrombocytopenia, diarrhea, fatigue, cough, pyrexia, upper respiratory tract infection, hypokalemia, muscle spasms, peripheral edema, constipation, back pain, dyspnea, peripheral neuropathy, hypertension, acute renal injury, heart failure, deep vein thrombosis, ischemic heart disease, and pulmonary embolism. KRd, combination therapy of carfilzomib, lenalidomide, and dexamethasone; Rd, combination therapy of lenalidomide and dexamethasone; RRMM, relapsed and refractory multiple myeloma; ^a^Historical comparators

The flow chart for subject selection process is shown in Fig. 2. Table 2 shows the baseline characteristics of the matched cohort, excluding patients with underlying heart failure. The median (range) follow-up period in the heart failure cohort was 121 (0–600) days in the KRd group and 153.5 (0–600) days in the Rd group. Before matching, the Rd group had a higher mean age compared to the KRd group. However, after matching, the mean ages of the KRd and Rd groups were more comparable with 66 (28–87) and 67 (25–86), respectively. The proportions of male subjects in the KRd and Rd groups were 53.38% and 55.34%, respectively. After propensity score matching, all covariates between the KRd and Rd groups were well balanced, with standardized differences less than 0.1, suggesting that potential confounding factors were adequately controlled. Supplementary Table 4 shows the distribution of important characteristics for each outcome variable after matching.

**Table 2:** Baseline characteristics of the KRd and Rd groups in the heart failure cohort before and after propensity score matching

	Before matching		Std. diff	After matching		Std. diff
	KRd (*n* = 927)	Rd (*n* = 966)		KRd (*n* = 562)	Rd (*n* = 562)	
Demographic characteristics						
Age—year, median (range)	63 (28–87)	71 (25–92)	−0.79	66 (28–87)	67 (25–86)	−0.01
Male sex, no. (%)	515 (55.56)	507 (52.48)	0.06	300 (53.38)	311 (55.34)	−0.04
Time since diagnosis—year, median (range)	2 (0–13.0)	2.4 (0–13.2)	−0.28	2.2 (0–13.0)	2.3 (0–12.9)	0
Number of prior regimens, median (range)	2 (1–7)	2 (1–8)	0.14	2 (1–7)	2 (1–7)	0.03
Current conditions, no. (%)						
Anemia	217 (23.41)	259 (26.81)	−0.08	135 (24.02)	140 (24.91)	−0.02
Neutropenia	86 (9.28)	45 (4.66)	0.18	27 (4.80)	32 (5.69)	−0.04
Thrombocytopenia	42 (4.53)	25 (2.59)	0.11	18 (3.20)	18 (3.20)	0
Diarrhea	41 (4.42)	46 (4.76)	−0.02	20 (3.56)	24 (4.27)	−0.04
Fatigue	8 (0.86)	0 (0)	0.13	0 (0)	0 (0)	0
Cough	359 (38.73)	357 (36.96)	0.04	212 (37.72)	220 (39.15)	−0.03
Pyrexia	187 (20.17)	167 (17.29)	0.07	108 (19.22)	109 (19.40)	0
Upper respiratory tract infection	265 (28.59)	268 (27.74)	0.02	152 (27.05)	157 (27.94)	−0.02
Hypokalemia	26 (2.80)	22 (2.28)	0.03	10 (1.78)	14 (2.49)	−0.05
Muscle spasms	6 (0.65)	12 (1.24)	−0.06	5 (0.89)	5 (0.89)	0
Peripheral edema	34 (3.67)	65 (6.73)	−0.14	24 (4.27)	26 (4.63)	−0.02
Constipation	195 (21.04)	207 (21.43)	−0.01	119 (21.17)	125 (22.24)	−0.03
Back pain	186 (20.06)	198 (20.50)	−0.01	108 (19.22)	103 (18.33)	0.02
Dyspnea	2 (0.22)	2 (0.21)	0	2 (0.36)	1 (0.18)	0.0345
Peripheral neuropathy	215 (23.19)	201 (20.81)	0.06	128 (22.78)	130 (23.13)	−0.01
Hypertension	234 (25.24)	366 (37.89)	−0.27	175 (31.14)	178 (31.67)	−0.01
Acute renal injury	32 (3.45)	29 (3.00)	0.03	17 (3.02)	14 (2.49)	0.03
Deep vein thrombosis	10 (1.08)	22 (2.28)	−0.09	8 (1.42)	9 (1.60)	−0.01
Ischemic heart disease	55 (5.93)	137 (14.18)	−0.28	46 (8.19)	39 (6.94)	0.05
Pulmonary embolism	7 (0.76)	10 (1.04)	−0.03	5 (0.89)	5 (0.89)	0
Plasma cell leukemia	8 (0.86)	6 (0.62)	0.03	3 (0.53)	4 (0.71)	−0.02
Other cancer	350 (37.76)	322 (33.33)	0.09	206 (36.65)	203 (36.12)	0.01
Infection	173 (18.66)	144 (14.91)	0.10	89 (15.84)	97 (17.26)	−0.04
Hepatitis	32 (3.45)	31 (3.21)	0.01	16 (2.85)	18 (3.20)	−0.02
Arrhythmia	8 (0.86)	19 (1.97)	−0.09	7 (1.25)	5 (0.89)	0.03
Graft versus host disease	2 (0.22)	1 (0.10)	0.03	1 (0.18)	1 (0.18)	0
Chronic kidney disease			0.17			0.09
CKD stages 1–3	10 (1.08)	17 (1.76)		7 (1.25)	11 (1.96)	
CKD stages 4–5	24 (2.59)	51 (5.28)		17 (3.02)	15 (2.67)	
CKD stage unspecified	25 (2.70)	39 (4.04)		17 (3.02)	24 (4.27)	
Dyslipidemia	288 (31.07)	361 (37.37)	−0.13	194 (34.52)	193 (34.34)	0
Diabetes	194 (20.93)	236 (24.43)	−0.08	131 (23.31)	128 (22.78)	0.01
Cerebrovascular disease	28 (3.02)	65 (6.73)	−0.17	20 (3.56)	24 (4.27)	−0.04
Peripheral vascular disease	33 (3.56)	63 (6.52)	−0.14	30 (5.34)	26 (4.63)	0.03
Renal disease	64 (6.90)	117 (12.11)	−0.18	44 (7.83)	52 (9.25)	−0.05
Liver disease	181 (19.53)	172 (17.81)	0.04	99 (17.62)	109 (19.40)	−0.05
Chronic obstructive pulmonary disease	163 (17.58)	196 (20.29)	−0.07	114 (20.28)	112 (19.93)	0.01
Gastrointestinal bleeding	7 (0.76)	11 (1.14)	−0.04	4 (0.71)	3 (0.53)	0.02
Fracture	90 (9.71)	105 (10.87)	−0.04	52 (9.25)	56 (9.96)	−0.02
Charlson comorbidity index, median (range)	4 (2–17)	5 (2–19)	−0.07	4 (2–16)	4 (2–15)	0.04
Prior therapies, no. (%)						
Stem cell transplantation	463 (49.95)	267 (27.64)	0.47	225 (40.04)	233 (41.46)	−0.03
Bortezomib	853 (92.02)	853 (88.3)	0.13	504 (89.68)	508 (90.39)	−0.02
Lenalidomide	41 (4.42)	18 (1.86)	0.15	22 (3.91)	15 (2.67)	0.07
Current medication use, no. (%)						
Chemotherapy	230 (24.81)	178 (18.43)	0.16	120 (21.35)	118 (21.00)	0.01
Antiplatelets	177 (19.09)	189 (19.57)	−0.01	92 (16.37)	93 (16.55)	0
Anticoagulants	74 (7.98)	86 (8.90)	−0.03	46 (8.19)	44 (7.83)	0.01
Beta-blockers	49 (5.29)	82 (8.49)	−0.13	37 (6.58)	30 (5.34)	0.05
Calcium channel blockers	173 (18.66)	272 (28.16)	−0.23	129 (22.95)	130 (23.13)	0
Angiotensin converting enzyme inhibitors	4 (0.43)	9 (0.93)	−0.06	2 (0.36)	1 (0.18)	0.03
Angiotensin receptor blockers	120 (12.94)	198 (20.5)	−0.20	94 (16.73)	97 (17.26)	−0.01
Aldosterone antagonists	8 (0.86)	11 (1.14)	−0.03	4 (0.71)	5 (0.89)	−0.02
Antiarrhythmic agents	2 (0.22)	10 (1.04)	−0.10	2 (0.36)	1 (0.18)	0.03
Cholesterol lowering medications	153 (16.5)	230 (23.81)	−0.18	116 (20.64)	118 (21.00)	−0.01
Antidiabetic drugs	132 (14.24)	178 (18.43)	−0.11	96 (17.08)	87 (15.48)	0.04
Other treatments, no. (%)						
Radiotherapy	18 (1.94)	8 (0.83)	0.10	8 (1.42)	8 (1.42)	0
Thoracentesis or paracentesis	4 (0.43)	1 (0.10)	0.06	1 (0.18)	1 (0.18)	0

*CKD* Chronic kidney disease; *KRd* combination therapy of carfilzomib, lenalidomide, and dexamethasone; *Rd* combination therapy of lenalidomide and dexamethasone; *Std. diff*, Standardized difference

### Risk of safety outcomes

The incidence and HR for each outcome variable between the KRd and the Rd group are shown in Table 3. The risks of all of the blood and lymphatic system adverse events including anemia (HR 1.55; 95% CI 1.21–1.99), neutropenia (HR 1.62; 95% CI 1.32–1.98), thrombocytopenia (HR 2.19; 95% CI 1.59–3.02), and some non-hematological adverse events including cough (HR 1.32; 95% CI 1.09–1.60), hypokalemia (HR 1.56; 95% CI 1.14–2.13), constipation (HR 1.53; 95% CI 1.19–1.95), hypertension (HR 1.58; 95% CI 1.15–2.17), and heart failure (HR 2.04; 95% CI 1.24–3.35) were significantly higher in the KRd group compared to the Rd group. Among the non-hematological adverse events, heart failure and hypertension showed the greatest increase in risks in the KRd group compared to those in the Rd group. Kaplan–Meier survival curves for each outcome variable are shown in Supplementary Fig. 1.

**Table 3:** Incidences and risks of adverse outcomes in KRd and Rd groups with contemporaneous and historical comparator

	Main analysis with contemporaneous comparator							Sensitivity analysis with historical comparator						
	KRd			Rd			HR (95% CI)	KRd			Rd			HR (95% CI)
	Events (n, %)	person-years	events/10 person-years	Events (n, %)	person-years	events/10 person-years		Events (n, %)	person-years	events/10 person-years	Events (n, %)	person-years	events/10 person-years	
Anemia	148 (32.5)	172	8.6	111 (24.3)	225	4.9	1.55 (1.21–1.99)	191 (31.2)	240	7.96	173 (28.3)	300	5.77	1.25 (1.02–1.53)
Neutropenia	221 (38.8)	198	11.2	160 (28.1)	264	6.1	1.62 (1.32–1.98)	316 (51.6)	256	12.34	263 (43)	327	8.04	1.36 (1.15–1.60)
Thrombocytopenia	111 (19.2)	263	4.2	56 (9.7)	313	1.8	2.19 (1.59–3.02)	175 (28.6)	355	4.93	117 (19.1)	411	2.85	1.58 (1.25–2.00)
Diarrhea	65 (11.2)	263	2.5	57 (9.8)	305	1.9	1.27 (0.89–1.81)	93 (15.2)	367	2.53	97 (15.8)	407	2.38	1.03 (0.78–1.37)
Fatigue	6 (1)	305	0.2	7 (1.1)	343	0.2	0.93 (0.31–2.76)	9 (1.5)	415	0.22	3 (0.5)	461	0.07	3.13 (0.85–11.55)
Cough	227 (57.8)	99	22.9	196 (49.9)	120	16.3	1.32 (1.09–1.60)	277 (56.8)	119	23.28	320 (65.6)	121	26.45	0.89 (0.76–1.05)
Pyrexia	89 (17.7)	214	4.2	79 (15.7)	252	3.1	1.22 (0.9–1.66)	110 (16.1)	294	3.74	153 (22.4)	310	4.94	0.73 (0.57–0.93)
Upper respiratory tract infection	131 (29.3)	159	8.2	153 (34.2)	173	8.8	0.92 (0.73–1.16)	162 (26.8)	213	7.61	236 (39.1)	211	11.18	0.67 (0.55–0.82)
Hypokalemia	97 (16.4)	265	3.7	67 (11.4)	310	2.2	1.56 (1.14–2.13)	130 (16.1)	370	3.51	123 (15.3)	422	2.91	1.11 (0.87–1.42)
Muscle spasms	8 (1.3)	309	0.3	10 (1.7)	343	0.3	0.86 (0.34–2.18)	16 (1.9)	403	0.4	20 (2.4)	434	0.46	0.81 (0.42–1.57)
Peripheral edema	36 (6.3)	275	1.3	48 (8.4)	303	1.6	0.82 (0.53–1.26)	52 (6.5)	381	1.36	53 (6.6)	431	1.23	1.07 (0.73–1.57)
Constipation	150 (33)	168	8.9	110 (24.2)	206	5.3	1.53 (1.19–1.95)	196 (30)	246	7.97	177 (27.1)	289	6.12	1.19 (0.97–1.46)
Back pain	67 (13.5)	210	3.2	76 (15.4)	243	3.1	0.98 (0.71–1.37)	96 (14)	286	3.36	133 (19.4)	321	4.14	0.77 (0.59–1)
Dyspnea	1 (0.2)	305	0	0 (0)	341	0	-	1 (0.1)	412	0.02	1 (0.1)	454	0.02	1.02 (0.06–16.31)
Peripheral neuropathy	60 (13.6)	205	2.9	49 (11.1)	225	2.2	1.3 (0.89–1.90)	79 (12.4)	289	2.73	83 (13.1)	321	2.59	0.98 (0.72–1.33)
Hypertension	94 (22.7)	174	5.4	64 (15.4)	205	3.1	1.58 (1.15–2.17)	130 (22.2)	248	5.24	86 (14.7)	286	3.01	1.62 (1.24–2.13)
Acute renal failure	41 (7.1)	282	1.5	28 (4.9)	320	0.9	1.53 (0.95–2.48)	53 (6.6)	385	1.38	46 (5.8)	428	1.07	1.18 (0.79–1.74)
Heart failure	44 (7.8)	267	1.6	24 (4.3)	318	0.8	2.04 (1.24–3.35)	50 (6.3)	376	1.33	30 (3.8)	431	0.7	1.8 (1.14–2.83)
Deep vein thrombosis	27 (4.5)	292	0.9	28 (4.7)	328	0.9	1.08 (0.63–1.83)	35 (4.2)	397	0.88	27 (3.3)	434	0.62	1.34 (0.81–2.22)
Ischemic heart disease	44 (8.1)	251	1.8	45 (8.3)	291	1.5	1.03 (0.68–1.56)	57 (7.4)	362	1.57	67 (8.7)	395	1.7	0.88 (0.62–1.26)
Pulmonary embolism	21 (3.5)	297	0.7	20 (3.3)	340	0.6	1.14 (0.62–2.10)	24 (2.9)	411	0.58	21 (2.5)	443	0.47	1.18 (0.66–2.13)

Sensitivity analysis was conducted with the historical comparator group who had used Rd before January 1, 2018. *CI* confidence interval; *HR* hazard ratio; *KRd* combination therapy of carfilzomib, lenalidomide, and dexamethasone; *Rd* combination therapy of lenalidomide and dexamethasone

### Sensitivity analyses

Table 3 shows the results of the first sensitivity analysis, which utilized a historical comparator group consisting of individuals who received Rd before January 1, 2018. The utilization of the historical comparator group resulted in an HR value that was more aligned with the null hypothesis. This finding indicates that the risks of adverse events were comparatively less distinct between the KRd and Rd groups in the sensitivity analysis compared to the original analysis. Unlike the original analysis, the first sensitivity analysis did not find significant differences in the risks of cough, hypokalemia, and constipation between the KRd and Rd groups. Additionally, pyrexia and upper respiratory tract infection occurred more frequently in the historical Rd group. However, for most of the other outcome variables, the direction and the statistical significance of the HR remained consistent in the first sensitivity analysis compared to those in the original analysis.

Supplementary Table 5 shows the results of the second sensitivity analysis, where the grace period was not limited to 60 days. The risk of peripheral neuropathy was not found to be significantly different between the KRd and Rd groups in original analysis. However, peripheral neuropathy occurred more frequently in the KRd group than in the Rd group in the sensitivity analysis. On the other hand, for all other outcome variables included in the second sensitivity analysis, the direction and statistical significance of the HR remained consistent with the original analysis. This indicates that the results for those outcome variables were robust and not substantially affected by the changes in the grace period.

## Discussion

In this study, the safety of carfilzomib, the medication used for treating RRMM, was evaluated using nationwide health insurance claims data from South Korea. The analysis employed the TTE method to evaluate the risk of various adverse events associated with carfilzomib treatment in an Asian population. The study findings showed a statistically significant increase in the risk of several adverse events when carfilzomib was used for treating RRMM. Specifically, the analysis showed a higher risk of anemia, neutropenia, thrombocytopenia, cough, hypokalemia, constipation, hypertension, and heart failure associated with carfilzomib in treating RRMM. These results emphasize the importance of considering the potential adverse events when utilizing carfilzomib in the management of RRMM. The use of nationwide health insurance claims data from South Korea and the application of the TTE method contribute to understanding the safety profile of carfilzomib in an Asian population.

In our study, the analysis revealed that the risks of hematologic adverse events including anemia, neutropenia, and thrombocytopenia, were significantly higher in the KRd group compared to the Rd group. These findings are consistent with previous RCTs (Dimopoulos et al. 2016; Hájek et al. 2017; Stewart et al. 2015) and observational studies (Imtiaz et al. 2021; Kawaji‐Kanayama et al. 2022; Rocchi et al. 2021) supporting the notion that carfilzomib treatment is associated with an increased risk of hematologic adverse events. The increased risks of anemia, neutropenia, and thrombocytopenia emphasize the need for the monitoring of hematologic adverse events when using carfilzomib.

The incidence of certain non-hematologic adverse events including cough, hypokalemia, constipation, hypertension, and heart failure was higher in the KRd group compared to the Rd group. These findings are consistent with several previous studies (Bishnoi et al. 2021; Dimopoulos et al. 2016; Fakhri et al. 2020; Hájek et al. 2017; Stewart et al. 2015; Waxman et al. 2018). In our study, hypokalemia events were of grade 2 or higher requiring the use of potassium supplements. Although hypokalemia does not present with symptoms, it holds clinical significance due to its potential to cause heart-related diseases such as electrocardiographic changes and cardiac arrhythmias (Kardalas et al. 2018). The exact mechanism by which carfilzomib induces hypokalemia has not been determined, however it is believed to involve the impact of carfilzomib on K^+^ channels and cell functioning (So et al. 2019). Additionally, the use of carfilzomib was associated with an increased risk of cardiovascular and pulmonary adverse events, particularly hypertension and heart failure (Bishnoi et al. 2021; Fakhri et al. 2020; Waxman et al. 2018). Cough was identified as a potential symptom of cardiopulmonary adverse events (Fakhri et al. 2020). In our study, the incidence of heart failure in the KRd group was higher (7.8%) compared to the Rd group (4.3%). These rates were slightly higher than those reported in the ASPIRE trial (KRd 6.4%, Rd 4.1%). Furthermore, the incidence of hypertension was also higher in our study (KRd 22.7%, Rd 15.4%) compared to the ASPIRE trial (KRd 14.3%, Rd 6.9%) (Stewart et al. 2015). These results might be due to the real-world setting of our study, which did not exclude patients with cardiovascular comorbidities and risk factors, in contrast to the ASPIRE trial (Stewart et al. 2015) which strictly excluded such patients. A previous observational study in Japan, which included 50 carfilzomib users, reported one case of heart failure and could not confirm an association between carfilzomib and heart failure (Kawaji‐Kanayama et al. 2022). In our current study, we conducted propensity score matching to ensure similarity between the KRd and Rd groups to emulate the design of the RCT using nationwide data in Korea. Our results were comparable to those reported in the ASPIRE trial (Stewart et al. 2015) and the subgroup analysis of RCTs including Asians (Dimopoulos et al. 2019). There were no discernible differences when comparing the outcomes between the Korean population and a non-Asian study population, which also indicated the robustness of our study.

Our findings support needs for monitoring of cardiovascular adverse events during treatment of carfilzomib. The 2022 European Society of Cardiology guidelines recommend regular monitoring of blood pressure, natriuretic peptides, and echocardiography at baseline and throughout therapy, particularly during the first six cycles (Lyon et al. 2022). These guidelines also recognize the limited availability of studies to establish an optimal monitoring schedule for cardiovascular adverse events, and they highlight a low level of evidence. Additionally, previous research utilizing data from the FDA’s adverse event reporting system indicated that cardiovascular adverse events tend to occur early, with a median onset time of 41 days (Zhai et al. 2021). This underlines the importance of closely monitoring cardiovascular adverse events within the first 40 days of treatment. Further research is necessary to determine the optimal frequency of monitoring for effectively preventing cardiovascular adverse events.

This study had several strengths that contribute to its robustness and applicability. Firstly, it was designed as a systematic study utilizing the TTE method, which involved careful selection of appropriate case and comparator regimens. This approach effectively enhanced the ability to draw causal inference, and mitigated the risk of immortal time bias. However, it is important to note that the absence of core elements like randomization limits the study’s ability to fully emulate an ideal clinical trial. To address the unmeasured confounding bias, we performed two sensitivity analyses. Although most of the outcomes agreed with the results of the original analysis, pyrexia, and upper respiratory tract infection showed higher risk in the historical comparator group, which was contrary to the ASPIRE trial (Stewart et al. 2015). We thought the reason for the difference might be attributed to the change in the clinical environments, specifically infection prevention measures had become more intensive in recent times. Overall, these results of our study should be considered as supportive evidence in clinical practice. Secondly, the utilization of claims data from South Korea allowed for generating evidence of carfilzomib safety in an Asian population. Previous RCTs predominantly included individuals of Caucasian ethnicity, making the inclusion of Asian individuals in this study particularly valuable. Thirdly, this study’s exclusion criteria were not overly restrictive, enhancing the generalizability of the findings to a wide range of clinical practice. This flexibility allows for greater relevance and applicability of the results to real-world settings. Finally, this study was strengthened by the inclusion of a large sample size of patients with RRMM by utilizing a nationwide database in Korea. The comprehensive coverage of approximately 97% of Korean population under the National Health Insurance system facilitated the thorough screening of 4,580 RRMM patients and the collection of all relevant medical data. By utilizing this real-world evidence methodology and the Korean insurance claims database, the study provided meaningful insights into the safety profile of carfilzomib in the context of the rare diseases RRMM.

This study also had few limitations that should be acknowledged. Firstly, the precise dosing information of carfilzomib could not be fully captured due to the nature of the claims database. The claims database lacks specific details regarding the exact dosing of injectable drugs like carfilzomib, as they are charged based on bottle units. Therefore, our study could not identify the reduction of carfilzomib dosage for managing intolerance. Additionally, the cycles of treatment regimens could not be identified using the claims database. However, the HR calculations were adjusted for exposure time to account for the lack of cycle information. Secondly, the severity of adverse events was not assessed. Additional information, including symptoms and laboratory test results, is required to determine the grading of adverse events. However, such information is not available in claims databases. Thirdly, our study primarily focused on evaluating the safety outcomes of carfilzomib, utilizing claims data, especially since its clinical efficacy has been demonstrated in the ASPIRE trial (Stewart et al. 2015). Consequently, we did not evaluate the real-world effectiveness of carfilzomib or the risk–benefit ratio for individual patients; instead, our focus was on the drug’s safety profile due to the notable adverse effects reported. As more long-term data become available, analyzing overall survival and quality of life in patients who have adverse reactions will be crucial, providing valuable insights that could enhance clinical decision-making. Fourthly, it was challenging to differentiate whether the observed events after drug administration were exacerbations of the underlying disease or adverse drug events. To mitigate this potential bias, the cohort entry date was set as the first prescription date of either KRd or Rd therapy, and patients with outcome variables before the index date were excluded from the analysis. This approach aimed to minimize the likelihood of attributing pre-existing conditions as adverse drug events. Despite these limitations, our study employed robust methods such as TTE and appropriate exclusion criteria to overcome these challenges and provide valuable insights into the real-world safety profile of carfilzomib.

## Conclusions

The results of this study indicate that observational studies utilizing the TTE method can provide valuable estimates of results comparable to RCTs. In patients with RRMM in Korea, the incidence of hematologic adverse events, including anemia, neutropenia, and thrombocytopenia were significantly higher in the KRd group compared to that in the Rd group. These results indicate the importance of monitoring for hematologic adverse events when administering carfilzomib. Furthermore, the study revealed a significantly higher incidence of non-hematologic adverse events such as constipation, cough, hypokalemia, hypertension, and heart failure in the KRd group compared to the Rd group. These safety outcomes were consistent with previous RCTs, suggesting that carfilzomib can be used in Asians populations with appropriate monitoring for tolerability.

## Supplementary Information

Below is the link to the electronic supplementary material.

Supplementary file1 (PDF 453 KB)

## Declarations

### Data availability

The datasets generated and analyzed in the current study are not publicly available because the Korea Health Insurance Review and Assessment Service only allows access to restricted researchers who have been approved for a specific study protocol. Researchers can apply for the data from the Korea Health Insurance Review and Assessment Service on research purposes.
