# Supplementary material for: Target trial emulation of carfilzomib safety among patients with relapsed/refractory multiple myeloma using a nationwide observational data in Korea
Source: J Cancer Res Clin Oncol. 2024 May 20;150(5):266. doi: 10.1007/s00432-024-05800-8 (PMC11106147; doi:10.1007/s00432-024-05800-8)
Supplement: Supplementary file 1 — Supplementary file1 (PDF 453 KB) [file 432_2024_5800_MOESM1_ESM.pdf]

Supplementary Material

Target trial emulation of carfilzomib safety among patients with relapsed/refractory multiple myeloma using a nationwide observational data in Korea

Journal of Cancer Research and Clinical Oncology

Hyun Kyung Lee, Ha Young Jang, In-Wha Kim, Jung Mi Oh*

*** Correspondence:**

Jung Mi Oh

College of Pharmacy and Research Institute of Pharmaceutical Sciences, Seoul National University, Seoul, South Korea

E-mail: jmoh@snu.ac.kr

Supplementary Table 1 Exclusion criteria according to adverse outcome variables

SOC	Outcome variables	Exclusion criteria
Blood and lymphatic system disorders	Anemia	• Diagnosed with anemia within 60 days prior to the index date<br>• Prescribed an erythropoietin stimulating agent within 60 days before the index date
	Neutropenia	• Diagnosed with neutropenia within 60 days prior to the index date<br>• Prescribed colony stimulating factor within 60 days before the index date
	Thrombocytopenia	• Diagnosed with thrombocytopenia within 60 days prior to the index date<br>• Received platelet transfusion within 60 days prior to the index date
Cardiac disorders	Heart failure	• Diagnosed with heart failure within 4 months prior to the index date
	Ischemic heart disease	• Diagnosed with ischemic heart disease within 4 months prior to the index date
Gastrointestinal disorders	Diarrhea	• Diagnosed with diarrhea within 60 days before the index date<br>• Prescribed loperamide within 60 days before the index date
	Constipation	• Diagnosed with constipation within 60 days before the index date
General disorders	Fatigue	• Diagnosed with fatigue within 60 days prior to the index date
	Pyrexia	• Diagnosed with pyrexia within 60 days prior to the index date
	Peripheral edema	• Diagnosed with peripheral edema within 60 days before the index date
Infections	Upper respiratory tract infection	• Diagnosed with upper respiratory tract infection within 60 days prior to the index date
Metabolic and nutrition disorders	Hypokalemia	• Diagnosed with hypokalemia within 60 days prior to the Index date<br>• Prescribed oral potassium supplements within 60 days before the index date
Musculoskeletal and connective tissue disorders	Muscle spasms	• Diagnosed with muscle spasms within 60 days prior to the Index date
	Back pain	• Diagnosed with low back pain within 60 days before the index date.
Nervous system disorders	Peripheral neuropathy	• Diagnosed with peripheral neuropathy within 60 days before the index date<br>• Prescribed gabapentin, pregabalin, or duloxetine within 60 days prior to the index date
Renal and urinary disorders	Acute renal failure	• Diagnosed with acute renal failure within 60 days before the index date
Respiratory, thoracic and mediastinal disorders	Cough	• Diagnosed with cough within 60 days prior to the index date<br>• Prescribed antitussive expectorants within 60 days before the index date
	Dyspnea	• Diagnosed with dyspnea within 60 days prior to the index date
	Pulmonary embolism	• Diagnosed with dyspnea within 60 days prior to the index date pulmonary embolism
Vascular disorders	Hypertension	• Diagnosed with hypertension within 60 days prior to the index date<br>• Prescribed antihypertensive drugs within 60 days prior to the index date
	Deep vein thrombosis	• Diagnosed with deep vein thrombosis within 60 days before the index date

SOC, system organ class

Supplementary Table 2 Operational definition of adverse outcome variables

SOC	variables	operational definition (disease: KCD-8, drug: ATC code, procedure: procedure code)	references
Blood and lymphatic system disorders	Anemia	disease OR drug<br>1. disease:<br>D50 (Iron deficiency anemia),<br>D51 (Vitamin B12 deficiency anemia),<br>D52 (Folate deficiency anemia,<br>D53 (Other nutritional anemia,<br>D59 (Acquired hemolytic anemia),<br>D60 (Acquired pure red cell aplasia [erythroblastopenia]),<br>D61 (Other aplastic anemia),<br>D62 (Acute posthemorrhagic anemia<br>D63 (Anemia in chronic diseases classified elsewhere),<br>D64 (Other anemias)<br>2. drug: B03X (Other antianemic preparations)	PMID: 30946433,<br>PMID: 33494720,<br>NCCN Guidelines: Cancer- and Chemotherapy-Induced anemia
	Neutropenia	disease OR drug<br>1. disease: D70 (Agranulocytosis)<br>2. drug: L03AA (Colony stimulating factors)	PMID: 30946433,<br>PMID: 33494720
	Thrombocytopenia	disease OR procedure<br>1. disease:<br>D691 (Qualitative platelet defects),<br>D693 (Idiopathic thrombocytopenic purpura),<br>D694 (Other primary thrombocytopenia),<br>D695 (Secondary thrombocytopenia),<br>D696 (Thrombocytopenia, unspecified)<br>2. procedure:<br>X2071 (Platelet rich plasma for whole blood 320ml),<br>X2072 (Platelet Rich Plasma for Whole Blood 400ml),<br>X2081 (Platelet Concentrate for Whole Blood 320ml),<br>X2082 (Platelet Concentrate for Whole Blood 400ml),<br>X2516 (Human platelets, washed),<br>X2121 (Leukocyte Filtered Packed Platelet Concentrate for Whole Blood 320ml),<br>X2122 (Leukocyte Filtered Packed Platelet Concentrate for Whole Blood 400ml)	PMID: 33494720
Cardiac disorders	Heart failure	disease: I50 (Heart failure)	EUPAS30748,<br>PMID: 32725984,<br>PMID: 33813836
	Ischemic heart disease	disease:<br>I20 (Angina pectoris),<br>I21 (Acute myocardial infarction),<br>I22 (Subsequent myocardial infarction),<br>I23 (Certain current complications following acute myocardial infarction),<br>I24 (Other acute ischemic heart diseases),<br>I25 (Chronic ischemic heart disease)	PMID: 33813836,<br>PMID: 32442285
Gastrointestinal disorders	Diarrhea	disease OR drug<br>1. disease: K591 (Functional diarrhea)<br>2. drug: loperamide	PMID: 33494720

SOC	variables	operational definition (disease: KCD-8, drug: ATC code, procedure: procedure code)	references
	Constipation	disease: K590 (Constipation)	-
General disorders	Fatigue	disease: R53 (Malaise and fatigue)	PMID: 33494720
	Pyrexia	disease: R50 (Fever of other and unknown origin)	-
	Peripheral edema	disease: R60 (Edema, NEC)	-
Infections	Upper respiratory tract infection	disease:<br>J00 (Acute nasopharyngitis [common cold]),<br>J01 (Acute sinusitis),<br>J02 (Acute pharyngitis),<br>J03 (Acute tonsillitis),<br>J04 (Acute laryngitis and tracheitis),<br>J05 (Acute obstructive laryngitis [croup] and epiglottitis),<br>J06 (Acute upper respiratory infections of multiple and unspecified sites),<br>J31 (Chronic rhinitis, nasopharyngitis and pharyngitis),<br>J32 (Chronic sinusitis),<br>J34 (Other disorders of nose and nasal sinuses),<br>J36 (Peritonsillar abscess),<br>J37 (Chronic laryngitis and laryngotracheitis),<br>J38 (Diseases of vocal cords and larynx, NEC)	-
Metabolic and nutrition disorders	Hypokalemia	disease OR drug<br>1. disease: E876 (Hypokalemia)<br>2. drug: A12BA01 (potassium chloride)	-
Musculoskeletal and connective tissue disorders	Muscle spasms	disease:<br>R252 (Cramp and spasm),<br>R29 (Other symptoms and signs involving the nervous and musculoskeletal systems),<br>R56 (Convulsions, NEC)	-
	Back pain	disease: M545 (Low back pain)	-
Nervous system disorders	Peripheral neuropathy	disease AND drug<br>1. disease:<br>G60 (Hereditary and idiopathic neuropathy),<br>G61 (Inflammatory polyneuropathy),<br>G62 (Other polyneuropathies),<br>G63 (Polyneuropathy in diseases classified elsewhere),<br>M541 (Radiculopathy),<br>M792 (Neuralgia and neuritis, unspecified)<br>2. drug: gabapentin, pregabalin, duloxetine	PMID: 33494720
Renal and urinary disorders	Acute renal injury	disease: N17 (Acute renal failure)	PMID: 30946433, EUPAS26595
Respiratory, thoracic and mediastinal disorders	Cough	disease OR drug<br>1. disease: R05 (Cough)<br>2. drug: R05 (Cough and cold preparations)	-
	Dyspnea	disease: R060 (Dyspnea)	PMID: 33107944, PMID: 31721140
	Pulmonary embolism	disease:<br>I260 (Pulmonary embolism with mention of acute cor pulmonale),<br>I269 (Pulmonary embolism without mention of acute cor pulmonale)	PMID: 31395174, PMID: 33494720

SOC	variables	operational definition (disease: KCD-8, drug: ATC code, procedure: procedure code)	references
Vascular disorders	Hypertension	disease AND drug<br>1. disease:<br>I10 (Essential(primary) hypertension),<br>I11 (Hypertensive heart disease),<br>I12 (Hypertensive renal disease),<br>I13 (Hypertensive heart and renal disease),<br>I15 (Secondary hypertension)<br>2. drug (ATC code):<br>C02 (Antihypertensives),<br>C07 (Beta blocking agents),<br>C08 (Calcium channel blockers),<br>C09 (Agents acting on the renin-angiotensin system)	PMID: 32725984
	Deep vein thrombosis	disease:<br>I82 (Other venous embolism and thrombosis),<br>I802 (vein thrombosis NOS),<br>I803 (Embolism or thrombosis of lower extremity NOS)	PMID: 31395174,<br>PMID: 33494720

KCD, Korean Standard Classification of Diseases; SOC, system organ class

Supplementary Table 3 Operational definition of covariates other than outcome variables

variables	operational definition (disease: KCD-8, drug: ATC code, procedure: procedure code)	screening period
Age	Age of the index date	-
Sex	Male or female	-
Period after diagnosis of multiple myeloma	Number of days since 'C90 (Multiple myeloma and malignant plasma cell neoplasms)' diagnosis	No limit
Number of previous treatment regimens	The previous treatment regimens were defined as follows. It was defined as regimen when all combination drugs were prescribed on the same day. If a smaller regimen was included in the larger regimen, only the large regimen was considered when the interval between administration days was less than 30 days. For example, if the date of administration of Rd regimen was between 30 days before and 30 days after the KRd administration period, it was defined as only KRd therapy.<br><br>Daratumumab: daratumumab containing regimen<br>VTDPACE: bortezomib + thalidomide + dexamethasone + cyclophosphamide + etoposide + cisplatin + doxorubicin (containing DTPACE, DCEP, VTD, VCD, PAD, TCD, VD, TD, BPLD, CD)<br>DTPACE: dexamethasone + thalidomide + cisplatin + doxorubicin + cyclophosphamide + etoposide (containing DCEP, TCD, TD, CD)<br>DCEP: dexamethasone + cyclophosphamide + etoposide + cisplatin (containing CD)<br>EDAP: etoposide + dexamethasone + cytarabine + cisplatin<br>VTD: bortezomib + thalidomide + dexamethasone (containing VD, TD)<br>VMP: bortezomib + melphalan + prednisolone (containing MP)<br>MPT: melphalan + prednisolone + thalidomide (containing MP, TP)<br>PCD: pomalidomide + cyclophosphamide + dexamethasone (containing POMDEX, CD)<br>VAD: vincristine + doxorubicin + dexamethasone<br>VRD: bortezomib + lenalidomide + dexamethasone (containing VD, RD)<br>VCD: bortezomib + cyclophosphamide + dexamethasone (containing VD, CD)<br>PAD: bortezomib + doxorubicin + dexamethasone (containing VD, BPLD)<br>TCD: thalidomide + cyclophosphamide + dexamethasone (containing TD, CD)<br>VD: bortezomib + dexamethasone<br>TD: thalidomide + dexamethasone<br>POMDEX: pomalidomide + dexamethasone<br>KD: carfilzomib + dexamethasone<br>BPLD: bortezomib + doxorubicin<br>BENDAPD: bendamustine + prednisolone<br>CD: cyclophosphamide + dexamethasone<br>MP: melphalan + prednisolone<br>MD: melphalan + dexamethasone<br>TP: thalidomide + prednisolone<br>CP: cyclophosphamide + prednisolone	No limit
Waldenstrom's macroglobulinemia	disease:<br>C880 (Waldenstrom macroglobulinemia)	60 days
Plasma cell myeloma	disease:<br>C901 (Plasma cell leukemia)	60 days
Cancer	disease: C, not C90	3 years
Infections	drug: ATC code J, only intravenous drugs	60 days
Hepatitis	disease:<br>B16 (Acute hepatitis B),<br>B170 (Acute delta-(super)infection of hepatitis B carrier),<br>B180 (Chronic viral hepatitis B with delta- agent),	60 days

variables	operational definition (disease: KCD-8, drug: ATC code, procedure: procedure code)	screening period
	B181 (Chronic viral hepatitis B without delta- agent),<br>B171 (Acute hepatitis C),<br>B182 (Chronic viral hepatitis C)	
Arrhythmia	disease:<br>I47 (Paroxysmal tachycardia),<br>I49 (Other cardiac arrhythmias)	4 months
Graft-versus-host disease	disease: T860 (Bone-marrow transplant rejection)	60 days
Chronic renal failure	disease:<br>N181 (Chronic kidney disease, stage 1)<br>N182 (Chronic kidney disease, stage 2)<br>N183 (Chronic kidney disease, stage 3)<br>N184 (Chronic kidney disease, stage 4)<br>N185 (Chronic kidney disease, stage 5)<br>N189 (Chronic kidney disease, unspecified)	60 days
Dyslipidemia	disease: E78 (Disorders of lipoprotein metabolism and other lipidemias)	60 days
Diabetes	disease:<br>E11 (Type 2 diabetes mellitus),<br>E12 (Malnutrition-related diabetes mellitus),<br>E13 (Other specified diabetes mellitus),<br>E14 (Unspecified diabetes mellitus)	60 days
Cerebrovascular diseases	disease:<br>G45 (Transient cerebral ischemic attacks and related syndromes),<br>G46 (Vascular syndromes of brain in cerebro- vascular diseases(I60-I67)),<br>I60 (Subarachnoid hemorrhage),<br>I61 (Intracerebral hemorrhage),<br>I62 (Other nontraumatic intracranial hemorrhage),<br>I63 (Cerebral infarction),<br>I64 (Stroke, not specified as hemorrhage or infarction),<br>I65 (Occlusion and stenosis of precerebral arteries, not resulting in cerebral infarction),<br>I66 (Occlusion and stenosis of cerebral arteries, not resulting in cerebral infarction),<br>I67 (Other cerebrovascular diseases),<br>I68 (Cerebrovascular disorders in diseases classified elsewhere),<br>I69 (Sequelae of cerebrovascular disease),<br>H340 (Transient retinal artery occlusion)	60 days
Peripheral vascular diseases	disease:<br>I70 (Atherosclerosis),<br>I71 (Aortic aneurysm and dissection),<br>I731 (Thromboangiitis obliterans[Buerger]),<br>I738 (Other specified peripheral vascular diseases),<br>I739 (Peripheral vascular disease, unspecified),<br>I771 (Stricture of artery),<br>I790 (Aneurysm of aorta in diseases classified elsewhere),<br>I792 (Peripheral angiopathy in diseases classified elsewhere),<br>K551 (Chronic vascular disorders of intestine),<br>K558 (Other vascular disorders of intestine),<br>K559 (Vascular disorder of intestine, unspecified),<br>Z958 (Presence of other cardiac and vascular implants and grafts),<br>Z959 (Presence of cardiac and vascular implant and graft, unspecified)	60 days
Kidney diseases	disease:<br>I120 (Hypertensive renal disease with renal failure),	60 days

variables	operational definition (disease: KCD-8, drug: ATC code, procedure: procedure code)	screening period
	I131 (Hypertensive heart and renal disease with renal failure),<br>N030 (Chronic nephritic syndrome with minor glomerular abnormality),<br>N031 (Chronic nephritic syndrome with focal and segmental glomerular lesions),<br>N032 (Chronic nephritic syndrome with diffuse membranous glomerulonephritis),<br>N033 (Chronic nephritic syndrome with diffuse mesangial proliferative glomerulonephritis),<br>N034 (Chronic nephritic syndrome with diffuse endocapillary proliferative glomerulonephritis),<br>N035 (Chronic nephritic syndrome with diffuse mesangiocapillary glomerulonephritis),<br>N036 (Chronic nephritic syndrome with dense deposit disease),<br>N037 (Chronic nephritic syndrome with diffuse crescentic glomerulonephritis),<br>N038 (Other chronic nephritic syndrome),<br>N039 (Unspecified chronic nephritic syndrome),<br>N050 (Unspecified nephritic syndrome with minor glomerular abnormality),<br>N051 (Unspecified nephritic syndrome with focal and segmental glomerular lesions),<br>N052 (Unspecified nephritic syndrome with diffuse membranous glomerulonephritis),<br>N053 (Unspecified nephritic syndrome with diffuse mesangial proliferative glomerulonephritis),<br>N054 (Unspecified nephritic syndrome with diffuse endocapillary proliferative glomerulonephritis),<br>N055 (Unspecified nephritic syndrome with diffuse mesangiocapillary glomerulonephritis),<br>N056 (Unspecified nephritic syndrome with dense deposit disease),<br>N057 (Unspecified nephritic syndrome with diffuse crescentic glomerulonephritis),<br>N058 (Unspecified other nephritic syndrome),<br>N059 (Unspecified nephritic syndrome),<br>N18 (Chronic kidney disease),<br>N19 (Unspecified kidney failure),<br>N250 (Renal osteodystrophy),<br>Z490 (Persons encountering health services for preparatory care for dialysis),<br>Z491 (Persons encountering health services for extracorporeal dialysis),<br>Z492 (Persons encountering health services for other dialysis),<br>Z940 (Kidney transplant status),<br>Z992 (Dependence on renal dialysis)	
Liver diseases	disease:<br>B16 (Acute hepatitis B),<br>B17 (Other acute viral hepatitis),<br>B18 (Chronic viral hepatitis),<br>B19 (Unspecified viral hepatitis),<br>K70 (Alcoholic liver disease),<br>K71 (Toxic liver disease),<br>K72 (Hepatic failure, NEC),<br>K73 (Chronic hepatitis, NEC),<br>K74 (Fibrosis and cirrhosis of liver),<br>K75 (Other inflammatory liver diseases),<br>K76 (Other diseases of liver),<br>T864 (Liver transplant failure and rejection),<br>Z944 (Liver transplant status),<br>R17 (Unspecified jaundice)	60 days

variables	operational definition (disease: KCD-8, drug: ATC code, procedure: procedure code)	screening period
Chronic obstructive pulmonary diseases	disease:<br>J40 (Bronchitis, not specified as acute or chronic),<br>J41 (Simple and mucopurulent chronic bronchitis),<br>J42 (Unspecified chronic bronchitis),<br>J43 (Emphysema),<br>J44 (Other chronic obstructive pulmonary disease)	60 days
Gastrointestinal bleeding	disease:<br>K260 (Acute duodenal ulcer with hemorrhage),<br>K262 (Acute duodenal ulcer with both hemorrhage and perforation),<br>K264 (Chronic or unspecified duodenal ulcer with hemorrhage),<br>K266 (Chronic or unspecified duodenal ulcer with both hemorrhage and perforation),<br>K270 (Acute peptic ulcer, site unspecified with hemorrhage),<br>K272 (Acute peptic ulcer, site unspecified with both hemorrhage and perforation),<br>K274 (Chronic or unspecified peptic ulcer, site unspecified with hemorrhage),<br>K276 (Chronic or unspecified peptic ulcer, site unspecified with both hemorrhage and perforation),<br>K280 (Acute gastrojejunal ulcer with hemorrhage),<br>K282 (Acute gastrojejunal ulcer with both hemorrhage and perforation),<br>K284 (Chronic or unspecified gastrojejunal ulcer with hemorrhage),<br>K286 (Chronic or unspecified gastrojejunal ulcer with both hemorrhage and perforation),<br>K290 (Acute hemorrhagic gastritis),<br>K625 (Hemorrhage of anus and rectum),<br>K920 (Hematemesis),<br>K921 (Melena),<br>K922 (Gastrointestinal hemorrhage, unspecified)	60 days
Fracture	disease:<br>S02 (Fracture of skull and facial bones),<br>S12 (Fracture of neck),<br>S22 (Fracture of rib(s), sternum and thoracic spine),<br>S32 (Fracture of lumbar spine and pelvis),<br>S42 (Fracture of shoulder and upper arm),<br>S52 (Fracture of forearm),<br>S62 (Fracture at wrist and hand level),<br>S72 (Fracture of femur),<br>S82 (Fracture of lower leg, including ankle),<br>S92 (Fracture of foot, except ankle),<br>T02 (Fractures involving multiple body regions),<br>T08 (Fracture of spine, level unspecified),<br>T10 (Fracture of upper limb, level unspecified),<br>T12 (Fracture of lower limb, level unspecified)	60 days
Charlson comorbidity index	CCI = cci1 + cci2 + cci3 + cci4 + cci5 + cci6 + cci7 + cci8 + cci9 + cci10 + 2*cci11 + 2*cci12 + 2*cci13 + 2*cci14 + 3*cci15 + 6*cci16 + 6*cci17<br>cci1: I21, I22, I252<br>cci2: I43, I50, I099, I110, I130<br>cci3: I70, I71, I731, I738, I739<br>cci4: G45, G46, I60, I61, I62, I63, I64, I65, I66, I67, I68<br>cci5: F00, F01, F02, F03, G30<br>cci6: J40, J41, J42, J43, J44, J45, J46, J47, J60, J61<br>cci7: M05, M06, M32, M33, M34<br>cci8: K25, K26, K27, K28	1 year

variables	operational definition (disease: KCD-8, drug: ATC code, procedure: procedure code)	screening period
	cci9: B18, K73, K74, K700, K701<br>cci10: E100, E101, E106, E108, E109, E110, E111<br>cci11: E102, E103, E104, E105, E107, E112, E113, E114, E115, E117, E122, E123, E124, E125, E127, E132, E133, E134, E135, E137, E142, E143, E144, E145, E147<br>cci12: G81, G82, G041, G114, G80<br>cci13: N18, N19, I120, I131, N032<br>cci14: C00, C01, C02, C03, C04, C05, C06, C07, C08<br>cci15: I850, I859, I864, I982, K704, K711<br>cci16: C77, C78, C79, C80<br>cci17: B20, B21, B22, B24	
Hematopoietic stem cell transplantation	procedure:<br>X5137 (Hemopoietic Cell Transplantation-Cord Blood(Allogenic))<br>X5131 (Hemopoietic Cell Transplantation-Bone Marrow(Allogenic))<br>X5132 (Hemopoietic Cell Transplantation-Bone Marrow(Autologous))<br>X5133 (Hemopoietic Cell Transplantation-Blood-Derived Hematopoietic Progenitor Cell(Allogenic))<br>X5134 (Hemopoietic Cell Transplantation-Blood-Derived Hematopoietic Progenitor Cell(Autologous))<br>X5135 (Hemopoietic Cell Transplantation-Cord Blood(Allogenic))<br>X5136 (Hemopoietic Cell Transplantation-Cord Blood(Autologous))	No limit
Bortezomib	drug:<br>L01XG01 (bortezomib)	No limit
Chemotherapy	Same as the definitions of treatment regimens	60 days
Antiplatelets	drug:<br>B01AC (platelet aggregation inhibitors excl. heparin)	60 days
Anticoagulants	drug:<br>B01AA (vitamin k antagonists)<br>B01AB (heparin group)<br>B01AD (enzymes)<br>B01AE (direct thrombin inhibitors)<br>B01AF (direct factor xa inhibitors)<br>B01AX (other antithrombotic agents)	60 days
Beta-blockers	drug:<br>C07 (beta blocking agents)	60 days
Calcium channel blockers	drug:<br>C07FB (beta blocking agents and calcium channel blockers)<br>C08 (calcium channel blockers)<br>C09BB (ace inhibitors and calcium channel blockers)<br>C09DB (angiotensin ii receptor blockers (arbs) and calcium channel blockers)	60 days
Angiotensin converting enzyme inhibitors	drug:<br>C09A (ace inhibitors, plain)<br>C09B (ace inhibitors, combinations)	60 days
Angiotensin receptor blockers	drug:<br>C09C (angiotensin ii receptor blockers (arbs), plain)<br>C09D (angiotensin ii receptor blockers (arbs), combinations)	60 days
Aldosterone inhibitors	drug:<br>C03D (potassium-sparing agents)	60 days
Antiarrhythmic agents	drug:<br>C01B (antiarrhythmics, class i and iii)	60 days
Cholesterol lowering drugs	drug:<br>C10 (lipid modifying agents)	60 days

variables	operational definition (disease: KCD-8, drug: ATC code, procedure: procedure code)	screening period
	A10BH52 (gemigliptin and rosuvastatin)	
Antidiabetic drugs	drug:<br>A10 (drugs used in diabetes)	60 days
Radiation therapy	procedure:<br>HD053 (Radiotherapy Teletherapy-High Energy-Single Port)<br>HD054 (Radiotherapy Teletherapy-Low Energy-Paralled Opposed Ports)<br>HD055 (Radiotherapy Teletherapy-Middle Energy-Paralled Opposed Ports)<br>HD056 (Radiotherapy Teletherapy-High Energy-Paralled Opposed Ports)<br>HD057 (Radiotherapy Rotational Irradiation-Low Energy)<br>HD058 (Radiotherapy Rotational Irradiation-Middle Energy)<br>HD059 (Radiotherapy Rotational Irradiation-High Energy)<br>HD051 (Radiotherapy Teletherapy-Low Energy-Single Port)<br>HD052 (Radiotherapy Teletherapy-Middle Energy-Single Port)<br>HZ271 (Radiotherapy Intensity Modulated Radiation Therapy)<br>HD160 (Radiotherapy Ocular tumor brachytherapy treatment(Ruthenium-106))	60 days
Thoracentesis or paracentesis	procedure:<br>C8040 (Thoracentesis)<br>C8050 (Paracentesis, Abdominal Paracentesis)	60 days

Supplementary Table 4 Baseline characteristics of important covariates after propensity score matching for each adverse reaction cohort

characteristics	Cohort1: Anemia		Cohort2: Neutropenia		Cohort3: Thrombocytopenia	
	KRd (n=456)	Rd (n=456)	KRd (n=569)	Rd (n=569)	KRd (n=577)	Rd (n=577)
Age - yr, median (range)	67 (37–87)	67 (25–85)	67 (32–87)	67 (25–85)	67 (28–87)	67 (25–88)
Male sex, no. (%)	247 (54.2)	243 (53.3)	311 (54.7)	303 (53.3)	305 (52.9)	307 (53.2)
Time since diagnosis - yr, median (range)	2.3 (0–13)	2.3 (0–12.9)	2.2 (0–13)	2.1 (0–13.2)	2.3 (0–13)	2.1 (0–12.9)
Number of prior regimens, median (range)	2 (1–6)	2 (1–7)	2 (1–6)	2 (1–8)	2 (1–6)	2 (1–8)
Neuropathy at baseline, no.(%)	88 (19.3)	100 (21.9)	121 (21.3)	125 (22)	122 (21.1)	131 (22.7)
Prior therapies, no.(%)						
Transplant	183 (40.1)	189 (41.5)	224 (39.4)	232 (40.8)	232 (40.2)	227 (39.3)
Bortezomib	405 (88.8)	406 (89)	517 (90.9)	517 (90.9)	518 (89.8)	528 (91.5)
Lenalidomide	17 (3.7)	12 (2.6)	16 (2.8)	11 (1.9)	19 (3.3)	13 (2.3)

characteristics	Cohort4: Diarrhea		Cohort5: Fatigue		Cohort6: Cough	
	KRd (n=581)	Rd (n=581)	KRd (n=609)	KRd (n=581)	Rd (n=581)	KRd (n=609)
Age - yr, median (range)	67 (37–87)	67 (25–87)	67 (28–87)	67 (37–87)	67 (25–87)	67 (28–87)
Male sex, no. (%)	306 (52.7)	316 (54.4)	334 (54.8)	306 (52.7)	316 (54.4)	334 (54.8)
Time since diagnosis - yr, median (range)	2.2 (0–12.9)	2.1 (0–12.9)	2.1 (0.1–13)	2.2 (0–12.9)	2.1 (0–12.9)	2.1 (0.1–13)
Number of prior regimens, median (range)	2 (1–6)	2 (1–8)	2 (1–7)	2 (1–6)	2 (1–8)	2 (1–7)
Neuropathy at baseline, no.(%)	122 (21)	128 (22)	138 (22.7)	122 (21)	128 (22)	138 (22.7)
Prior therapies, no.(%)						
Transplant	235 (40.5)	232 (39.9)	243 (39.9)	235 (40.5)	232 (39.9)	243 (39.9)
Bortezomib	520 (89.5)	529 (91.1)	546 (89.7)	520 (89.5)	529 (91.1)	546 (89.7)
Lenalidomide	19 (3.3)	15 (2.6)	16 (2.6)	19 (3.3)	15 (2.6)	16 (2.6)

Supplementary Table 4 Baseline characteristics of important covariates after propensity score matching for each adverse reaction cohort (continued)

characteristics	Cohort7: Pyrexia		Cohort8: Upper respiratory tract infection		Cohort9: Hypokalemia	
	KRd (n=502)	KRd (n=447)	Rd (n=447)	KRd (n=447)	Rd (n=447)	KRd (n=609)
Age - yr, median (range)	67.5 (39–87)	67 (37–87)	67 (31–88)	67 (37–87)	67 (31–88)	67 (28–87)
Male sex, no. (%)	276 (55)	239 (53.5)	240 (53.7)	239 (53.5)	240 (53.7)	334 (54.8)
Time since diagnosis - yr, median (range)	2.1 (0–13)	2 (0–13)	2 (0–12.9)	2 (0–13)	2 (0–12.9)	2.1 (0.1–13)
Number of prior regimens, median (range)	2 (1–6)	2 (1–5)	2 (1–7)	2 (1–5)	2 (1–7)	2 (1–7)
Neuropathy at baseline, no.(%)	108 (21.5)	100 (22.4)	108 (24.2)	100 (22.4)	108 (24.2)	138 (22.7)
Prior therapies, no.(%)						
Transplant	185 (36.9)	163 (36.5)	165 (36.9)	163 (36.5)	165 (36.9)	243 (39.9)
Bortezomib	457 (91)	408 (91.3)	411 (92)	408 (91.3)	411 (92)	546 (89.7)
Lenalidomide	17 (3.4)	14 (3.1)	11 (2.5)	14 (3.1)	11 (2.5)	16 (2.6)

characteristics	Cohort10: Muscle spasms		Cohort11: Peripheral edema		Cohort12: Constipation	
	KRd (n=604)	Rd (n=604)	KRd (n=571)	KRd (n=604)	Rd (n=604)	KRd (n=571)
Age - yr, median (range)	67 (28–87)	67 (25–87)	67 (38–87)	67 (28–87)	67 (25–87)	67 (38–87)
Male sex, no. (%)	319 (52.8)	329 (54.5)	314 (55)	319 (52.8)	329 (54.5)	314 (55)
Time since diagnosis - yr, median (range)	2.1 (0–13)	2.1 (0–12.9)	2.1 (0–12.7)	2.1 (0–13)	2.1 (0–12.9)	2.1 (0–12.7)
Number of prior regimens, median (range)	2 (1–6)	2 (1–7)	2 (1–7)	2 (1–6)	2 (1–7)	2 (1–7)
Neuropathy at baseline, no.(%)	131 (21.7)	138 (22.9)	130 (22.8)	131 (21.7)	138 (22.9)	130 (22.8)
Prior therapies, no.(%)						
Transplant	235 (38.9)	229 (37.9)	223 (39.1)	235 (38.9)	229 (37.9)	223 (39.1)
Bortezomib	546 (90.4)	551 (91.2)	520 (91.1)	546 (90.4)	551 (91.2)	520 (91.1)
Lenalidomide	19 (3.2)	14 (2.3)	20 (3.5)	19 (3.2)	14 (2.3)	20 (3.5)

Supplementary Table 4 Baseline characteristics of important covariates after propensity score matching for each adverse reaction cohort (continued)

characteristics	Cohort13: Back pain		Cohort14: Dyspnea		Cohort15: Peripheral neuropathy	
	KRd (n=495)	Rd (n=495)	KRd (n=442)	KRd (n=442)	Rd (n=581)	KRd (n=609)
Age - yr, median (range)	67 (32–87)	67 (25–86)	67 (37-85)	67 (37-85)	67 (25–87)	67 (28–87)
Male sex, no. (%)	281 (56.8)	284 (57.4)	239 (54.1)	239 (54.1)	316 (54.4)	334 (54.8)
Time since diagnosis - yr, median (range)	2 (0.1–13)	2.1 (0–13.1)	2.2 (0.1-13)	2.2 (0.1-13)	2.1 (0–12.9)	2.1 (0.1–13)
Number of prior regimens, median (range)	2 (1–6)	2 (1–8)	2 (1-6)	2 (1-6)	2 (1–8)	2 (1–7)
Neuropathy at baseline, no.(%)	112 (22.6)	112 (22.6)	0 (0)	0 (0)	128 (22)	138 (22.7)
Prior therapies, no.(%)						
Transplant	178 (36)	191 (38.6)	178 (40.3)	178 (40.3)	232 (39.9)	243 (39.9)
Bortezomib	456 (92.1)	451 (91.1)	394 (89.1)	394 (89.1)	529 (91.1)	546 (89.7)
Lenalidomide	19 (3.8)	11 (2.2)	15 (3.4)	15 (3.4)	15 (2.6)	16 (2.6)

characteristics	Cohort16: Hypertension		Cohort17: Acute renal failure		Cohort18: Cardiac failure	
	KRd (n=415)	Rd (n=415)	KRd (n=576)	KRd (n=415)	Rd (n=415)	KRd (n=576)
Age - yr, median (range)	65 (28-87)	66 (25-86)	67 (35-87)	65 (28-87)	66 (25-86)	67 (35-87)
Male sex, no. (%)	226 (54.5)	232 (55.9)	306 (53.1)	226 (54.5)	232 (55.9)	306 (53.1)
Time since diagnosis - yr, median (range)	2.4 (0-13)	2.3 (0-12.9)	2.1 (0.1-13)	2.4 (0-13)	2.3 (0-12.9)	2.1 (0.1-13)
Number of prior regimens, median (range)	2 (1-6)	2 (1-7)	2 (1-6)	2 (1-6)	2 (1-7)	2 (1-6)
Neuropathy at baseline, no.(%)	92 (22.2)	92 (22.2)	119 (20.7)	92 (22.2)	92 (22.2)	119 (20.7)
Prior therapies, no.(%)						
Transplant	193 (46.5)	188 (45.3)	223 (38.7)	193 (46.5)	188 (45.3)	223 (38.7)
Bortezomib	365 (88)	368 (88.7)	517 (89.8)	365 (88)	368 (88.7)	517 (89.8)
Lenalidomide	14 (3.4)	10 (2.4)	17 (3)	14 (3.4)	10 (2.4)	17 (3)

Supplementary Table 4 Baseline characteristics of important covariates after propensity score matching for each adverse reaction cohort (continued)

characteristics	Cohort19: Deep vein thrombosis		Cohort20: Ischemic heart disease		Cohort21: Pulmonary embolism	
	KRd (n=601)	Rd (n=601)	KRd (n=544)	KRd (n=601)	Rd (n=601)	KRd (n=544)
Age - yr, median (range)	67 (37-87)	67 (25-87)	67 (37-87)	67 (37-87)	67 (25-87)	67 (37-87)
Male sex, no. (%)	325 (54.1)	333 (55.4)	297 (54.6)	325 (54.1)	333 (55.4)	297 (54.6)
Time since diagnosis - yr, median (range)	2.2 (0.1-13)	2.1 (0-12.9)	2.2 (0-13)	2.2 (0.1-13)	2.1 (0-12.9)	2.2 (0-13)
Number of prior regimens, median (range)	2 (1-7)	2 (1-7)	2 (1-6)	2 (1-7)	2 (1-7)	2 (1-6)
Neuropathy at baseline, no.(%)	132 (22)	135 (22.5)	124 (22.8)	132 (22)	135 (22.5)	124 (22.8)
Prior therapies, no.(%)						
Transplant	240 (39.9)	236 (39.3)	212 (39)	240 (39.9)	236 (39.3)	212 (39)
Bortezomib	543 (90.4)	546 (90.9)	488 (89.7)	543 (90.4)	546 (90.9)	488 (89.7)
Lenalidomide	19 (3.2)	14 (2.3)	15 (2.8)	19 (3.2)	14 (2.3)	15 (2.8)

KRd, combination therapy of carfilzomib, lenalidomide, and dexamethasone; Rd, combination therapy of lenalidomide and dexamethasone; yr, year; no., number

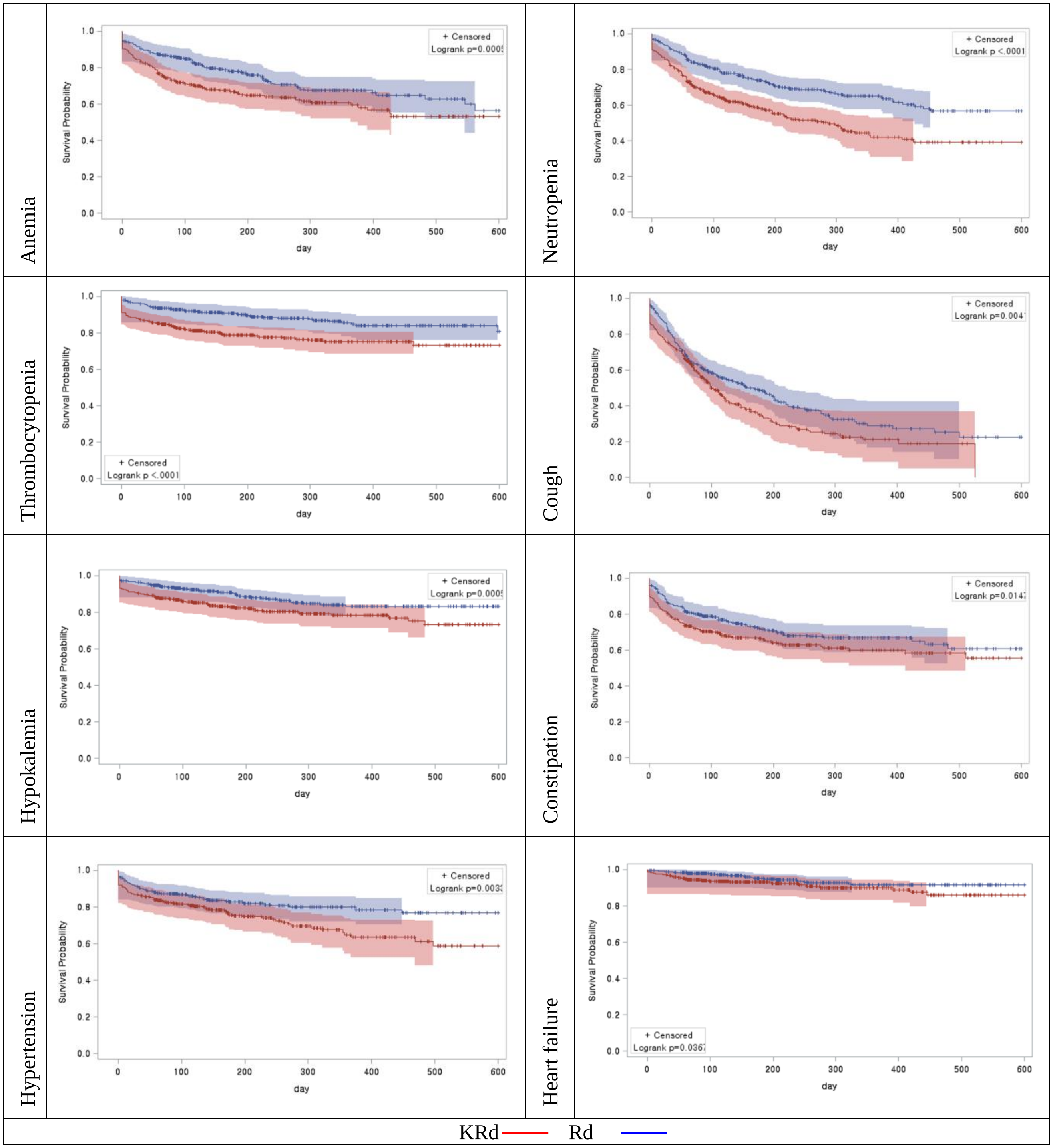

Supplementary Fig. 1 Kaplan-Meier survival curve for outcome variables with significantly higher risk in the KRd group compared to the Rd group. KRd, combination therapy of carfilzomib, lenalidomide, and dexamethasone; Rd, combination therapy of lenalidomide and dexamethasone

Supplementary Table 5 Sensitivity analysis in which the analysis was conducted without limiting the grace period

	KRd			Rd			HR (95% CI)
	events (n, %)	person-years	events/10 person-years	events (n, %)	person-years	events/10 person-years	
Anemia	162 (26.5)	202	8.02	122 (19.9)	270	4.52	1.57 (1.24–1.99)
Neutropenia	245 (40)	237	10.34	176 (28.8)	317	5.55	1.66 (1.36–2.01)
Thrombocytopenia	124 (20.3)	319	3.89	66 (10.8)	383	1.72	2.08 (1.54–2.80)
Diarrhea	81 (13.2)	322	2.52	73 (11.9)	368	1.98	1.24 (0.91–1.71)
Fatigue	6 (1)	385	0.16	8 (1.3)	419	0.19	0.82 (0.28–2.36)
Cough	242 (61.6)	107	22.62	220 (56)	135	16.3	1.29 (1.08–1.55)
Pyrexia	100 (19.9)	267	3.75	94 (18.7)	307	3.06	1.14 (0.86–1.51)
Upper respiratory tract infection	150 (33.6)	189	7.94	171 (38.3)	198	8.64	0.91 (0.73–1.14)
Hypokalemia	110 (18.6)	327	3.36	75 (12.7)	380	1.97	1.58 (1.18–2.12)
Muscle spasms	10 (1.7)	383	0.26	12 (2)	422	0.28	0.88 (0.38–2.04)
Peripheral edema	44 (7.7)	346	1.27	57 (10)	374	1.52	0.82 (0.55–1.22)
Constipation	164 (36.1)	207	7.92	122 (26.9)	247	4.94	1.50 (1.18–1.89)
Back pain	76 (15.4)	262	2.9	86 (17.4)	291	2.96	0.96 (0.70–1.30)
Dyspnea	1 (0.2)	382	0.03	1 (0.2)	421	0.02	1.28 (0.08–20.93)
Peripheral neuropathy	76 (17.2)	255	2.98	52 (11.8)	284	1.83	1.58 (1.11–2.25)
Hypertension	102 (24.6)	216	4.72	69 (16.6)	251	2.75	1.57 (1.16–2.13)
Acute renal failure	48 (8.3)	350	1.37	32 (5.6)	391	0.82	1.55 (0.99–2.43)
Heart failure	47 (8.4)	330	1.42	29 (5.2)	384	0.76	1.79 (1.12–2.84)
Deep vein thrombosis	29 (4.8)	367	0.79	34 (5.7)	403	0.84	0.91 (0.56–1.50)
Ischemic heart disease	46 (8.5)	316	1.46	50 (9.2)	356	1.4	0.96 (0.64–1.43)
Pulmonary embolism	23 (3.8)	374	0.61	26 (4.3)	420	0.62	0.94 (0.53–1.64)

CI, confidence interval; HR, hazard ratio; KRd, combination therapy of carfilzomib, lenalidomide, and dexamethasone; Rd, combination therapy of lenalidomide and dexamethasone
